# Bilateral decompressive craniotomy complicated by postoperative mycoplasma hominis epidural empyema and meningitis: A case report

**DOI:** 10.1097/MD.0000000000033745

**Published:** 2023-05-12

**Authors:** Lizhen Chen, Yue Lu, Jia Liu, Xiuzhong Zhang, Ke Wang

**Affiliations:** a Department of Clinical Laboratory, Chengdu Women’s and Children’s Central Hospital, School of Medicine, University of Electronic Science and Technology of China, Chengdu, China; b Department of Neurosurgery, The General Hospital of Western Theater Command PLA, Chengdu, China; c Key Laboratory of Advanced Technologies of Materials, Ministry of Education, School of Materials Science and Engineering, Southwest Jiaotong University, Chengdu, China.

**Keywords:** mycoplasma hominis, postoperative infection, traumatic brain injury

## Abstract

Postoperative intracranial mycoplasma hominis infection was a rare complication. Timely diagnosis was difficult due to its growth characteristics and nonspecific clinical symptoms.

**Patient concerns:**

A 52-year-old man underwent bilateral decompressive craniotomy for severe traumatic brain injury. On the seventeenth day after surgery, the patient developed an unexplained high fever. Empirical anti-infective therapy was ineffective, and the fever persisted. In addition, viscous pus oozed from the head incision. Empiric therapy was still ineffective, the fever persisted, and the culture result was negative. The lumbar puncture pressure was 150 mmH_2_O and the cerebrospinal fluid white blood cell count was 3600 × 10^6^/L, suggesting an intracranial infection.

**Diagnoses:**

Culture growth morphologically consistent with mycoplasma species was obtained from multiple specimens (scalp incision fluid and cerebrospinal fluid) and the identification of mycoplasma hominis was confirmed by 16S rDNA sequencing.

**Intervention:**

Targeted anti-infective therapy (Minocycline), change of fresh wound dressing, and continued lumbar cerebrospinal fluid drainage.

**Outcome:**

At the 3-month follow-up, the patient was still in the rehabilitation department of the local hospital for treatment, but there were no symptoms of intracranial infection.

**Lessons:**

Neurosurgeons should carefully examine postoperative incisions and be aware of the possibility of mycoplasma infection during clinical management.

## 1. Introduction

Postoperative central nervous system infection was a serious complication of cranial surgery with an incidence ranging from 0.7% to 8.9%.^[1]^ The most common bacteria were staphylococcus, but intracranial mycoplasma hominis infection was rare in patients. Here, we reported to a rare case of intracranial mycoplasma hominis infection after severe traumatic brain injury (TBI).

## 2. Case report

**Figure 1.**
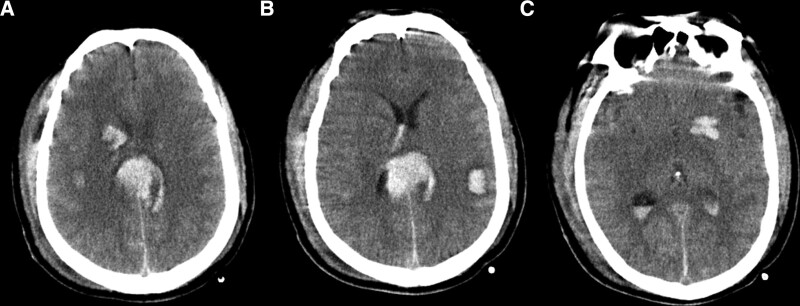
Preoperative cranial CT. CT = computed tomography.

A 52-year-old man presented with 12 hours of unconsciousness after a traffic accident. The patient had a history of appendectomy for 10 years. After the emergency treatment at the local hospital, the patient was transferred to our hospital. The computed tomography scan on admission showed diffuse brain swelling of the bilateral cerebral hemispheres and multiple cerebral hemorrhages (Fig. 1). Neurosurgical evaluation revealed a laceration (3 cm) in the superior part of the right head, and the patient had a Glasgow coma scale score of 5. He had a right upper extremity extension response with painful stimulation and miotic pupils (2.0/2.0, +/+).

The diagnosis was severe TBI. The patient accepted the implantation of an intracranial pressure (ICP) probe, and the initial ICP was 32.5 mm Hg after the use of mannitol. Therefore, the patient underwent emergency operation of bilateral decompressive craniotomy to control the ICP. Postoperative computed tomography showed better intracranial swelling than before (Figure S1, Supplemental Digital Content, http://links.lww.com/MD/I982). And the ICP fluctuated around 10 mm Hg after medical treatment.

On the fourth day, the sputum culture of the patient was aeruginosa. According to the antimicrobial susceptibility test results of drug, the patient accepted the piperacillin-sulbactam to anti-infection. Due to the persistent coma and pulmonary infection, the patient underwent tracheotomy on the seventh day after admission. With active treatment, the patient’s condition was stable.

On the seventeenth day, the patient developed an unexplained high fever (39.8℃). Because the lung condition improved (Figure S2, Supplemental Digital Content, http://links.lww.com/MD/I983), we performed blood culture, urine culture and lumbar puncture to exclude the causes of the fever. The lumbar puncture pressure was 150 mmH_2_O and the cerebrospinal fluid (CSF) white blood cell count was 3600 × 10^6^/L, suggesting an intracranial infection. The patient accepted empirical antibiotic treatment (vancomycin, 1000 mg/q12h) and continued lumbar CSF drainage.

While changing a fresh dressing on the wound, the viscous fluid (gray, odorless) leaked from the head incision. The fluid was sent to the microbiology laboratory for bacterial culture. However, the patient again had a high fever (39.1℃), and the CSF white blood cell count increased to 12,600 × 10^6^/L. Because the empiric therapy was still ineffective, the fever persisted, and the culture result was negative, we changed the antibiotics to meropenem (1 g/q8h) and linezolid (0.6 g/q12h). After 3 days of treatment, the CSF nucleated erythrocytes decreased, but low-grade fever still occurred.

**Figure 2.**
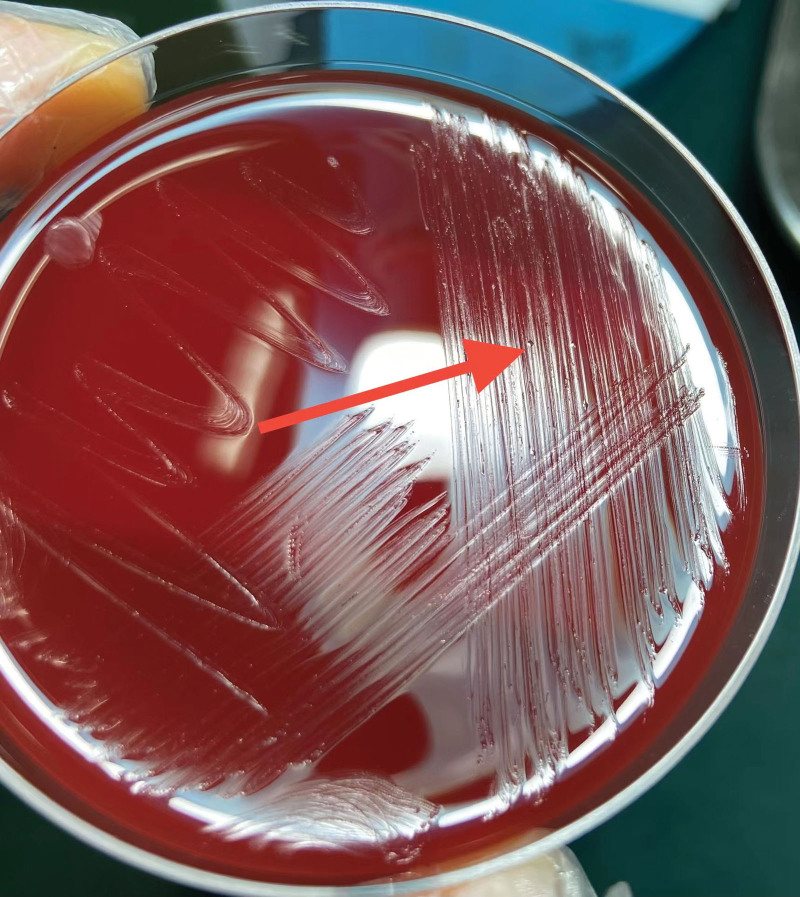
Pinpoint colonies (red arrow) visible on sheep blood agar.

**Figure 3.**
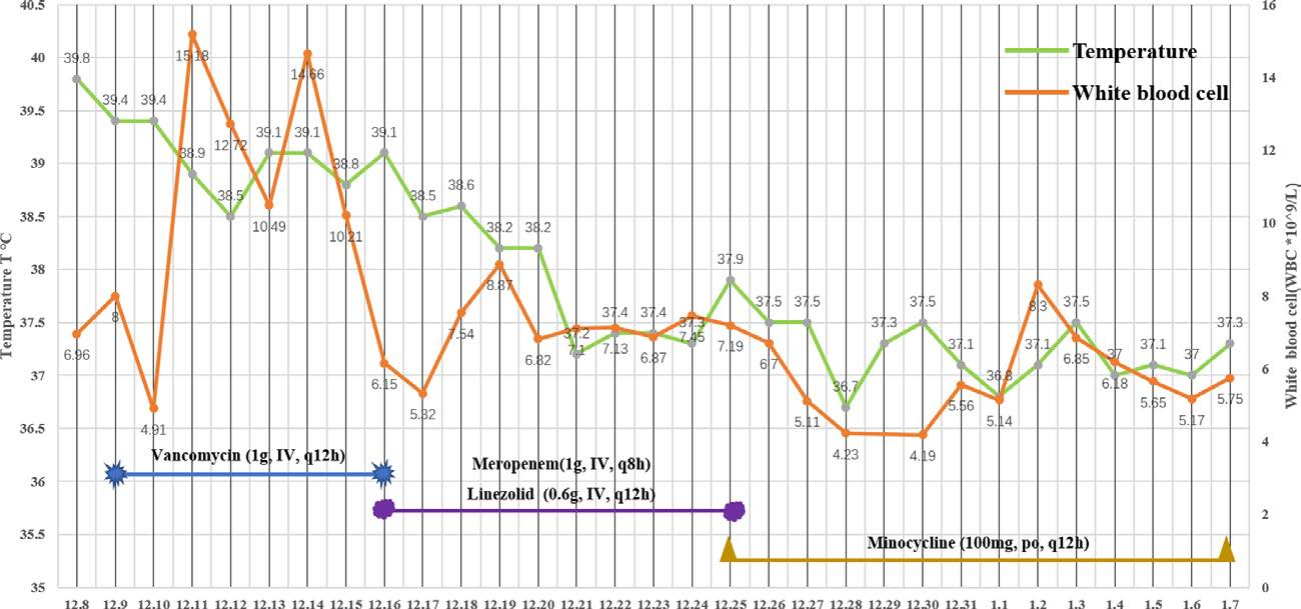
Relationship between the change of infection index (white blood cell, temperature) and the use of antibiotics during hospitalization.

Unexpectedly, possible punctate colonies were noted on sheep blood agar culture medium (Fig. 2), and Gram staining of the possible colonies was negative. Culture growth morphologically consistent with mycoplasma species was obtained from multiple specimens (scalp incision fluid and CSF) and the identification of mycoplasma hominis was confirmed by 16S rDNA sequencing. The patient was then started on minocycline (100 mg/bid). The fever and CSF nucleated erythrocytes recovered significantly 3 days after initiation of appropriate therapy (Fig. 3).

The patient was finally sent to the rehabilitation department to complete a 4-week course of minocycline 100 mg every 12 hours. After 3 months of follow-up, the patient was still in the rehabilitation department of the local hospital for treatment, but there were no symptoms of intracranial infection.

## 3. Discussion

Intracranial mycoplasma hominis infection was a rare postoperative complication. Risk factors included immunosuppression, malignancy, trauma, and manipulation or surgery of the genitourinary tract.^[2]^ In previous studies,^[3–6]^ patients with mycoplasma hominis cerebral infection usually presented with previous head trauma or neurosurgical procedures. In our case, risk factors such as severe TBI, surgery, decreased immunity, continuous urinary tube intubation and tracheal intubation were contributed to the extra-genital infections.

Fever was the common symptom of mycoplasma hominis infection, which was not nonspecific. However, the condition of the wound would be more meaningful. In the cases of previous studies,^[7–9]^ many patients presented with the redness, swelling and pain around the surgical site, liquid exudation from the wound. In our case, the gray, odorless, viscous subcutaneous fluid was discharged from the head incision. Therefore, we believed that postoperative incisions should be carefully examined, especially in postoperative patients with unexplained fever.

Due to the lack of a cell wall, slow growth of colonies, and difficulty in identification by Gram staining,^[2,3]^ the diagnosis of mycoplasma hominis by culture was usually delayed. In addition, mycoplasma hominis was always considered as contamination. Therefore, the timely diagnosis of intracranial mycoplasma hominis infection was challenging. With the development of new technologies for microbial species identification, such as Matrix-assisted Laser Desorption/ionization Time-of-Flight Mass Spectrometry and DNA or RNA sequencing, the time to microbiological diagnosis has been significantly reduced.^[10]^ We recommend that clinicians should be aware of the possibility of mycoplasma infection in equivocal intracranial infections, and remind the laboratory to perform more targeted testing.

The optimal therapeutic strategy for the treatment of mycoplasma hominis was targeted anti-infective therapy according to drug susceptibility testing.^[8]^ In addition, the wound dressing was required for patients with a history of open TBI or previous surgery. Moreover, lumbar puncture and continuous lumbar drainage of CSF were also important to detect and monitor of intracranial infection.

## 4. Conclusion

Although timely diagnosis was difficult and only the microbiologic evidence could confirm the diagnosis, unexplained postoperative fever, ineffective empiric treatment, and fluid exudation from the wound were considered. We recommended that the clinicians should be aware of the possibility of mycoplasma hominis involvement in postoperative infections, consider a timely change in antibiotic regimen, and perform more targeted testing to detect the potentially pathogenic organisms.

## Author contributions

**Conceptualization:** Lizhen Chen, Ke Wang.

Data curation: Lizhen Chen.

Investigation: Lizhen Chen, Yue Lu, Jia Liu, Xiuzhong Zhang.

Resources: Lizhen Chen.

Visualization: Lizhen Chen, Yue Lu.

Writing – original draft: Lizhen Chen, Yue Lu.

Writing – review & editing: Ke Wang.

## Supplementary Material




