# Supplementary figures and images for: Bilateral decompressive craniotomy complicated by postoperative mycoplasma hominis epidural empyema and meningitis: A case report
Source: Medicine (Baltimore). 2023 May 12;102(19):e33745. doi: 10.1097/MD.0000000000033745 (PMC10174387; doi:10.1097/MD.0000000000033745)

Supplementary Figure 1. Postoperative cranial CT

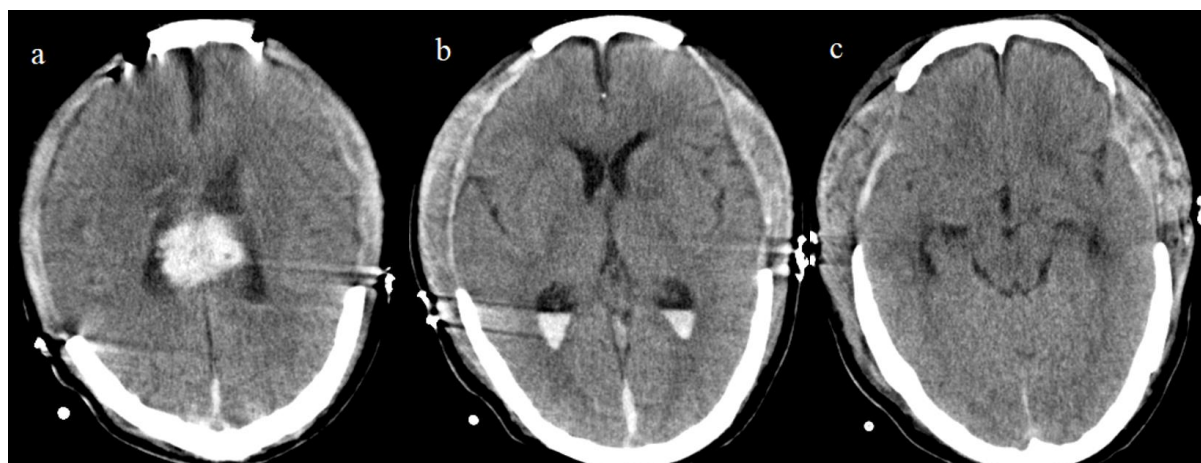

Supplement: Supplementary file 1 [file medi-102-e33745-s001.pdf]

Supplementary Figure 2. Chest CT in the presence of high fever

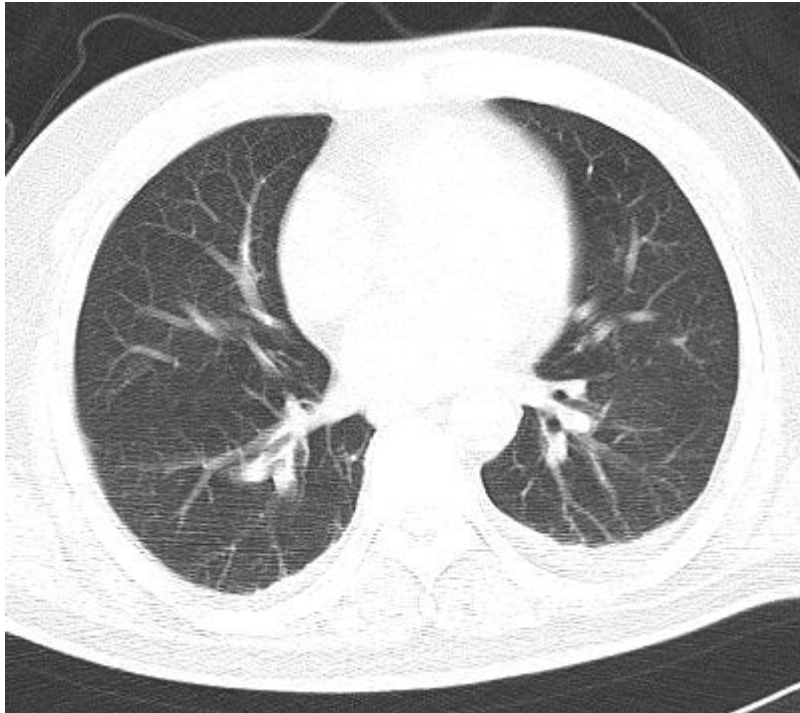

Supplement: Supplementary file 2 [file medi-102-e33745-s002.pdf]
